# Seasonal and environmental variation in volatile emissions of the New Zealand native plant *Leptospermum scoparium* in weed-invaded and non-invaded sites

**DOI:** 10.1038/s41598-020-68386-4

**Published:** 2020-07-16

**Authors:** Evans Effah, D. Paul Barrett, Paul G. Peterson, Murray A. Potter, Jarmo K. Holopainen, Andrea Clavijo McCormick

**Affiliations:** 1 School of Agriculture and Environment, Massey University, Tennent Drive, Palmerston North, 4474 New Zealand; 2 Manaaki Whenua - Landcare Research, Massey University, Riddet Road, Palmerston North, 4474 New Zealand; 3 Department of Environmental and Biological Sciences, University of Eastern Finland, Yliopistonranta 1 E, 70210 Kuopio, Finland

**Keywords:** Ecology, Invasive species

## Abstract

The New Zealand tea tree* Leptospermun scoparium* (mānuka) is widely known for the antimicrobial properties of its honey. Mānuka is native to New Zealand, growing in a range of environments, including the Central Volcanic Plateau of the North Island, where it is currently threatened by the spread of exotic invasive weeds such as heather (*Calluna vulgaris*) and Scotch broom (*Cytisus scoparius*). Here, we characterise for the first time the aboveground volatile organic compounds (VOCs) produced by mānuka in this area, during summer and winter seasons, in weed-invaded and non-invaded stands. We measured plant volatiles at four sites, each with a distinct combination of woody species: (1) conspecific stands of mānuka; (2) mānuka and another native species (*Dracophyllum subulatum*); and mānuka with one of two European invasive plants, (3) heather or (4) Scotch broom. We also quantified herbivore damage on target mānuka plants and analysed microclimatic variables (soil nutrients, air temperature and soil water content) to investigate their impact on volatile emissions. Our results reveal a strong seasonal effect on volatile emissions, but also significant differences between sites associated with biotic and abiotic changes partly driven by invasive plants. Overall, volatile emission rates from mānuka were typically lower at sites where invaders were present. We point to several factors that could contribute to the observed emission patterns and areas of interest for future research to provide a comprehensive understanding of VOC emissions in nature. Given the vital role of volatile compounds in plant communication, we also recommend future studies to be performed in multiple seasons, with larger sample sizes and more study sites to expand on these findings and explore the ecological impacts of changes in VOC emissions during plant invasion.

## Introduction

Volatile organic compounds (VOCs), like other plant secondary metabolites, are not directly involved in plant growth, development or reproduction^1^, but are vital to facilitate ecological interactions^2^. Such interactions include attracting key pollinators, repelling and deterring herbivores, attracting natural enemies of herbivores and beneficial microbes and shaping plant competition^3–5^. The composition of volatile blends depends on plant species, organ, developmental stage and physiological status of the emitting plant^6–9^. Some plants also adsorb their neighbour’s volatiles and passively re-release them^10,11^. However, volatile emission is also extremely plastic, with emissions varying in response to biotic and abiotic factors such as temperature, soil nutrients, herbivory or disease^12^. Species-specificity and environmental plasticity make VOCs an excellent source of information for other organisms, influencing the foraging behaviour of pollinators, herbivores and their natural enemies, and the competitive decisions of nearby plants^13,14^.

There is a growing body of evidence showing that VOC emissions depend on the species composition of neighbouring plants and associated environmental changes^15,16^. In plant invasion scenarios, it has been suggested that not only VOC emissions, but the response of the surrounding organisms to the emitted compounds, depends on whether the emitter is a native or exotic plant^17^. Moreover, previous studies suggest that exotic invasive species can recognise neighbours and adjust their metabolic responses accordingly. For example, the invasive species *Centaurea maculosa* accumulated higher levels of defence-related secondary metabolites and lower levels of primary metabolites when growing with conspecifics versus heterospecifics^18^.

The chemical behaviour of native plants in environments invaded by exotic species has rarely been studied. However, some native plants can outcompete or persist and coevolve with their invasive counterparts^19,20^, suggesting that natives are not necessarily passive during an exotic weed invasion.

The New Zealand tea tree *Leptospermun scoparium* (mānuka in Māori), is a member of the Myrtaceae family and is native to New Zealand and Australia. Mānuka is widely known for the antibacterial, antiviral and anti-inflammatory properties of honey produced from its nectar, which have been subject to extensive research^21,22^, suggesting the plant is a prolific producer of secondary metabolites. Mānuka is the most widely distributed, abundant, and environmentally tolerant member of New Zealand’s woody flora and is capable of enormous environmental plasticity^23^.

Mānuka is a mid-successional species but persists in some areas that do not support succession to climax forest. This includes some low nutrient and poorly drained sites on the Central Volcanic Plateau of the North Island in New Zealand^24^. Another mid-successional dominant native woody species adapted to similar environments in this area is *Dracohyllum subulatum*^25,26^. However, the survival of these and other low growing sub-alpine species on the Central Plateau is threatened by the spread of invasive plants such as heather (*Calluna vulgaris*) and broom (*Cytisus scoparius*). Both heather and broom are woody shrubs, introduced from Europe by early European settlers. Heather was intentionally introduced to Tongariro National Park (which lies within the Central Plateau) in 1912 and has now spread through most of the park and beyond its boundaries, while broom invasion only began in the 1960s and is not yet widespread^27,28^.

Analyses of the essential oils of mānuka indicate that this plant produces an array of secondary metabolites, being rich in sesquiterpenes^29,30^. New Zealand and Australian populations showed differences in their essential oils, with oils from Australian plants having significantly more monoterpenes than those in New Zealand^29^. However, the chemical ecology of mānuka remains largely unknown, and no previous study has investigated the plant’s volatile emissions (scents) or the factors accounting for their natural variation. Given the ecological importance of VOCs, this information is increasingly relevant as invasive weeds threaten the distribution of this species in its native range.

This study aimed to investigate the natural variation in volatile emissions of mānuka and to identify the factors regulating their emissions. This was done by selecting four sites on the North Island Central Plateau in New Zealand. Each site was distinct and characterised by the presence of mānuka in combination with conspecifics or one of three woody species; *Dracophyllum* (native), heather or broom (both exotics invaders). We measured VOCs in the headspace of target mānuka plants in both summer and winter, quantified herbivore damage on the target plants and collected microclimatic data (soil properties, environmental temperature and soil water content) from each site, to establish the effect of biotic and abiotic variables on VOC emissions.

## Material and methods

### Site description and experimental setup

The study was conducted from late November 2017 to September 2018, covering the summer and winter seasons. Four study sites (≥ 50 m × 50 m per site) with distinct plant combinations were selected in the Waiouru Military Training Area without manipulating any variables (Supplementary Table S1). Mānuka and *Dracophyllum* are common native woody perennials occurring naturally in the area, while both heather and broom were introduced from Europe. The sites differed in the dominant woody perennials present, with one site having predominantly mānuka plants (henceforth referred to as Mānuka – Mānuka or MM), another having a combination of predominantly mānuka and *Dracophyllum* (Mānuka – *Dracophyllum* or MD). The third site had a combination of predominantly mānuka and heather (Mānuka – Heather or MH), and the fourth a combination of predominantly mānuka and broom (Mānuka – Broom or MB) (Supplementary Table S1). Five replicates consisting of similar-sized mānuka plants were selected at each site. At each site, the positioning of the randomly chosen replicates covered ~ 25 m × 13 m of the respective sites (Supplementary Table S1), with about 0.5 m between target paired plants. During each season, data were collected from all sites and VOCs were collected from the same target mānuka plants.

### Measuring volatile emissions of mānuka

Aboveground VOCs of mānuka plants of similar size and phenology were collected at each site using the ‘push–pull’ headspace sampling technique and analysed following the protocol described in a previous study^16^. A similar amount of foliage of sampled plants was enclosed in new oven bags, and carbon filtered air simultaneously pushed into the bags through a PTFE tube (1.70 L/min) and pulled out (1.20 L/min) through another tube using a portable PVAS22 pump (Volatile Assay Systems Rensselaer NY). Volatiles in the headspace air were trapped onto a collection filter containing 30 mg HayeSep Q adsorbent (Volatile Assay Systems Rensselaer NY) inserted into the pull tube. VOCs were simultaneously collected from different sites at a time to minimise the effect of collection time. Volatiles from each sampled plant were collected for 2 h, after which the enclosed foliage was excised and oven-dried at 60 °C for 72 h to calculate emissions per dry weight.

Volatile collection filters were eluted using 200 µL of 95% hexane with 10 ng/mL nonyl acetate (C_11_H_22_O_2_) (Sigma Aldrich) and collected samples analysed using gas chromatography coupled to mass spectrometry. The GC–MS operation conditions and identification of compounds followed the same protocol described in^16^. VOCs were measured from the same target plants in summer (6–13 December 2017) and winter (26 August to 11 September 2018) under similar meteorological conditions.

### Examining herbivore damage on mānuka

Using a handheld magnifying glass, visible herbivore damage was examined on the same foliage used during the VOC collections. The number of damage marks on foliage was counted and divided by the dry weight (g) of foliage to estimate damage counts/g as described in^16^.

### Microclimatic measurements

To measure the soil properties of study sites, we took 20 soil cores (15 cm deep × 3 cm diameter) from each (i.e. 4 cores around each paired plants). We measured the fresh weight of soil and determined soil water content (SWC) after oven drying at 40 °C until constant weight. Dried soil from each site was then homogenised to represent the average for respective sites and used for nutrient analyses. Soil pH, total carbon (C), total nitrogen (N), potassium (K), calcium (Ca), magnesium (Mg) and sodium (Na), and soil temperature were measured as described in^16^.

Ambient air temperature was recorded by installing temperature data loggers (Tinytag, Gemini) 50 cm above ground level at each site 10 days prior to VOC measurements and collected on the last day of VOC measurements for each season.

### Data analysis

All statistical analyses were performed using R (version 3.6.2).

Major volatile classes were transformed by log10x + 1 and compared between the two sampling seasons using linear models (“lm” function in R). Before performing linear models, total monoterpenoids and sesquiterpenoids rates were normalised to standard temperature (30 °C) using an empirically derived coefficient (0.09) as recommended by Guenther and colleagues^31^.

The composition of volatile blends produced by mānuka plants was compared between the four sites using a permutational multivariate analysis of variance (PERMANOVA)^32^. PERMANOVA was performed using the vegan package. When there were significant differences between the sites, multiple comparisons were performed using the “pairwise.adonis” function and similarity percentage analysis (SIMPER) used to identify the volatile compounds accounting for the differences between sites^33^. The patterns in VOC emissions between sites were visualised using non-metric multidimensional scaling (NMDS), also with the vegan package. Both PERMANOVA and NMDS were based on Bray–Curtis dissimilarities using square root transformed VOCs data.

Herbivory, soil and ambient temperatures and SWC data were analysed using ANOVA or non-parametric Kruskal–Wallis, and when significant, followed by the Tukey HSD and Mann Whitney tests respectively for multiple comparisons.

We then investigated the potential effects of environmental variables on the VOCs selected through SIMPER using PERMANOVA based on Euclidean distances. PERMANOVA with Euclidean distance produced the classical univariate *F*-statistic, but robust to the assumption of normality and *P* values obtained by permutation^34,35^. Each model had one response variable (one volatile compound) and environmental factors (soil nitrogen, soil water content, herbivory and ambient temperature) as predictors. These predictors were selected based on previous reports on their effects on biogenic volatile organic compounds emission^7,12,36^. All response variables were square root transformed before modelling.

## Results

### Seasonal variation in volatile emissions

**Figure 1:**
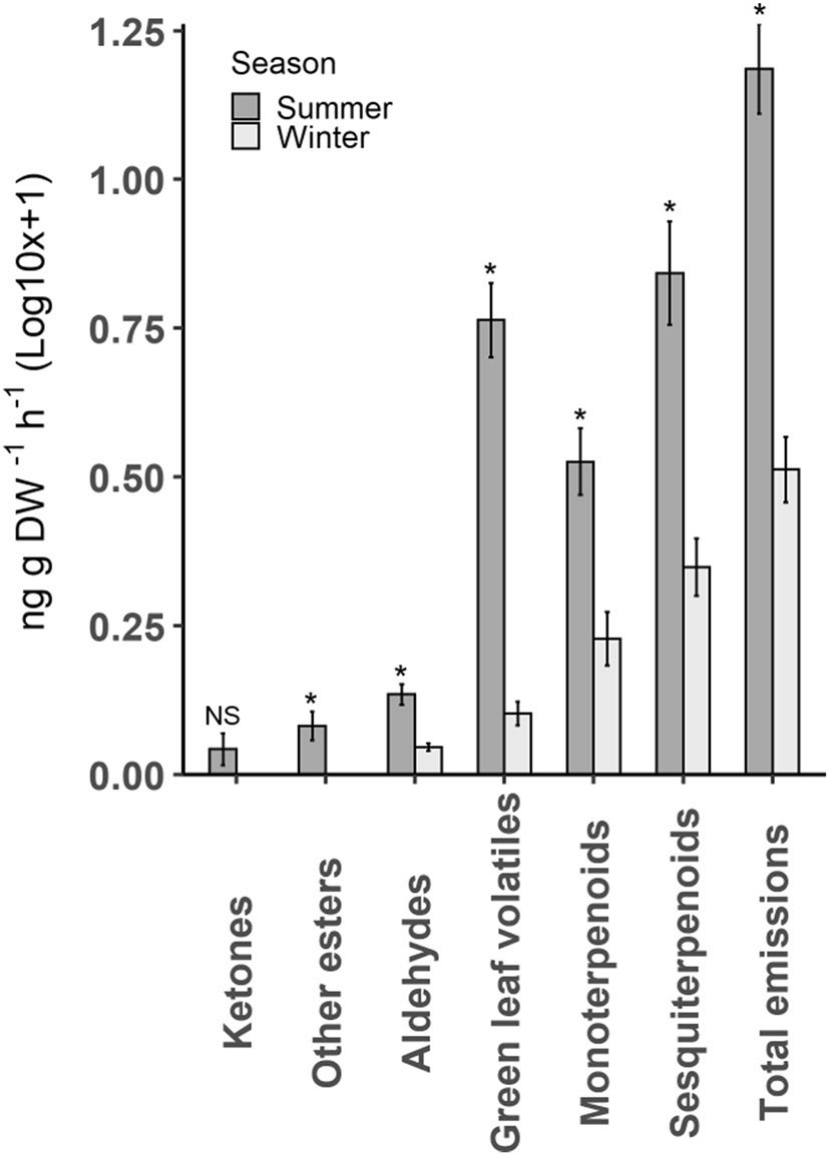
VOC classes for mānuka between the two sampling seasons (n = 20 for each season). Bars show mean ± SE of respective chemical classes. Asterisks (*) indicate a significant difference in emission between seasons and ‘NS’ means non-significant difference.

More volatile compounds were identified from the headspace of mānuka in summer (51 compounds) than in winter (34). VOCs identified from mānuka were classified into their respective chemical groups (Supplementary Table S4 and S5) and compared between the two seasons using linear models. The results show significantly lower emissions in winter for green leaf volatiles (*F*_1,38_ = 103.40, *P* < 0.001), sesquiterpenoids (*F*_1,38_ = 24.91, *P* < 0.001), monoterpenoids (*F*_1,38_ = 17.03, *P* < 0.001), aldehydes (*F*_1,38_ = 23.02, *P* < 0.001), other esters (*F*_1,38_ = 11.23, *P* = 0.002) and total volatile emissions (*F*_1,38_ = 52.33, *P* < 0.001, Fig. 1).

### Site variation in volatile emissions

**Figure 2:**
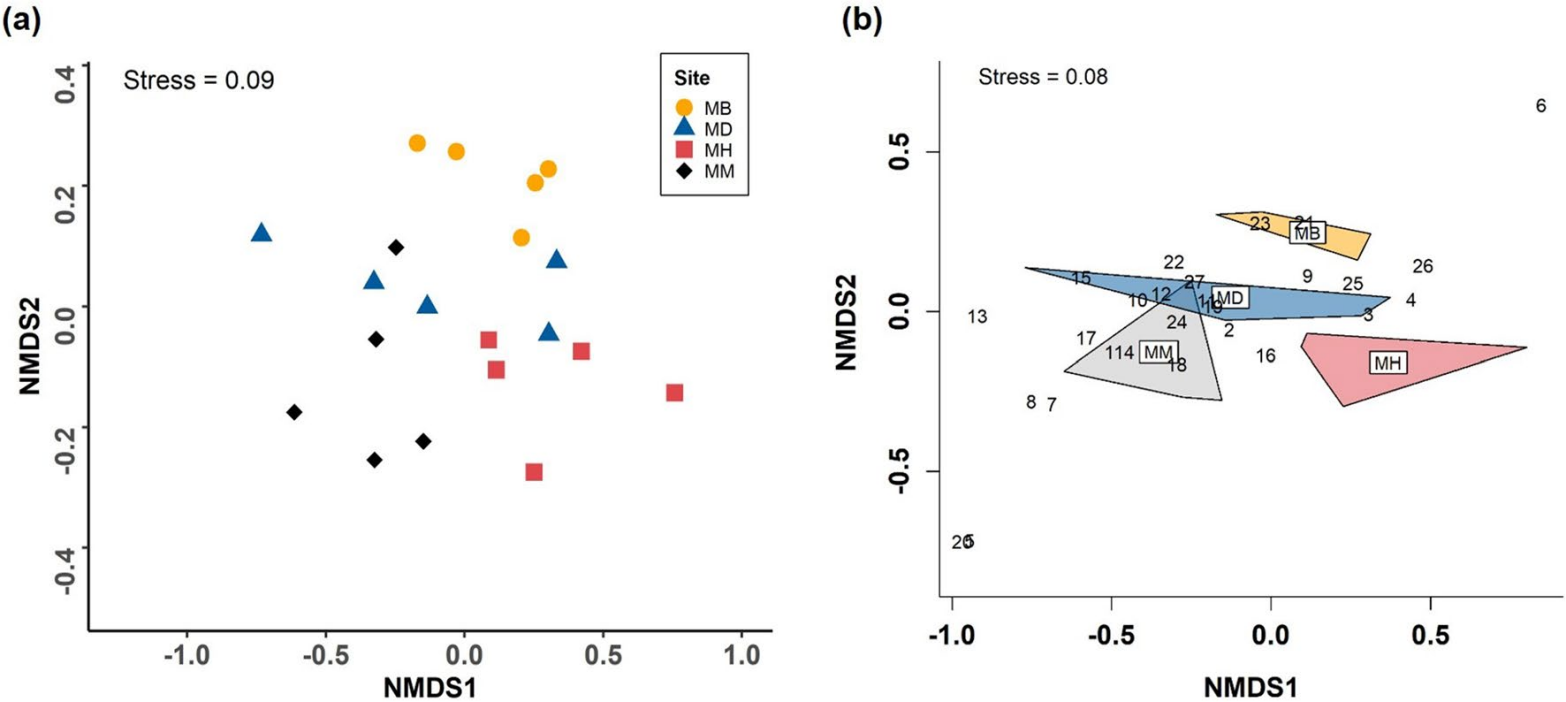
NMDS plots for VOCs emitted by mānuka at four different sites in summer. (**a**) Based on all the 51 VOCs identified from mānuka and (**b**) based on 27 VOCs with high contributions selected through SIMPER. The numbers on the graph represent the following compounds; (1) (*E*)-β-caryophyllene, (2) (*Z*)-3-hexenol, (3) (*Z*)-3-hexenyl acetate, (4) (*Z*)-β-ocimene, (5) (*Z*,*E*)-α-farnesene, (6) 2-heptanone, (7) 3-methyl-1-butanol acetate, (8) 2-methyl-1-butanol acetate, (9) aromadendrene, (10) cadinadiene-1,4, (11) calamenene, (12) copaene, (13) germacrene D, (14) humulene, (15) isoledene, (16) lemonol, (17) linalool, (18) o-cymene, (19) α-cubebene, (20) (*E*,*E*)-α-farnesene, (21) α-pinene, (22) α-selinene, (23) β-chamigrene, (24) β-elemene, (25) β-myrcene, (26) β-pinene, (27) β-selinene. Mānuka – Broom (MB), Mānuka – *Dracophyllum* (MD), Mānuka – Heather (MH), Mānuka – Mānuka (MM).

In summer, we detected significant variations in the volatile profile of mānuka between the four sites (PERMANOVA; Pseudo-*F* = 3.71, *P* < 0.001, Fig. 2). Volatile composition was significantly different between the conspecific stands and the mānuka – heather (Pseudo-*F* = 7.40, *P* = 0.012) or mānuka – broom (Pseudo-*F* = 7.49, *P* = 0.006, Fig. 2) sites. Difference was also significant between the sites where mānuka occurs with the two invasive plants (Pseudo-*F* = 3.62, *P* = 0.009, Fig. 2). Variations between the mānuka – *Dracophyllum* site and other sites were not significant (Fig. 2, Supplementary Table S2). The similarity percentage analysis revealed that 27 volatile compounds accounted for the observed pattern in volatile composition (Fig. 2b).

**Figure 3:**
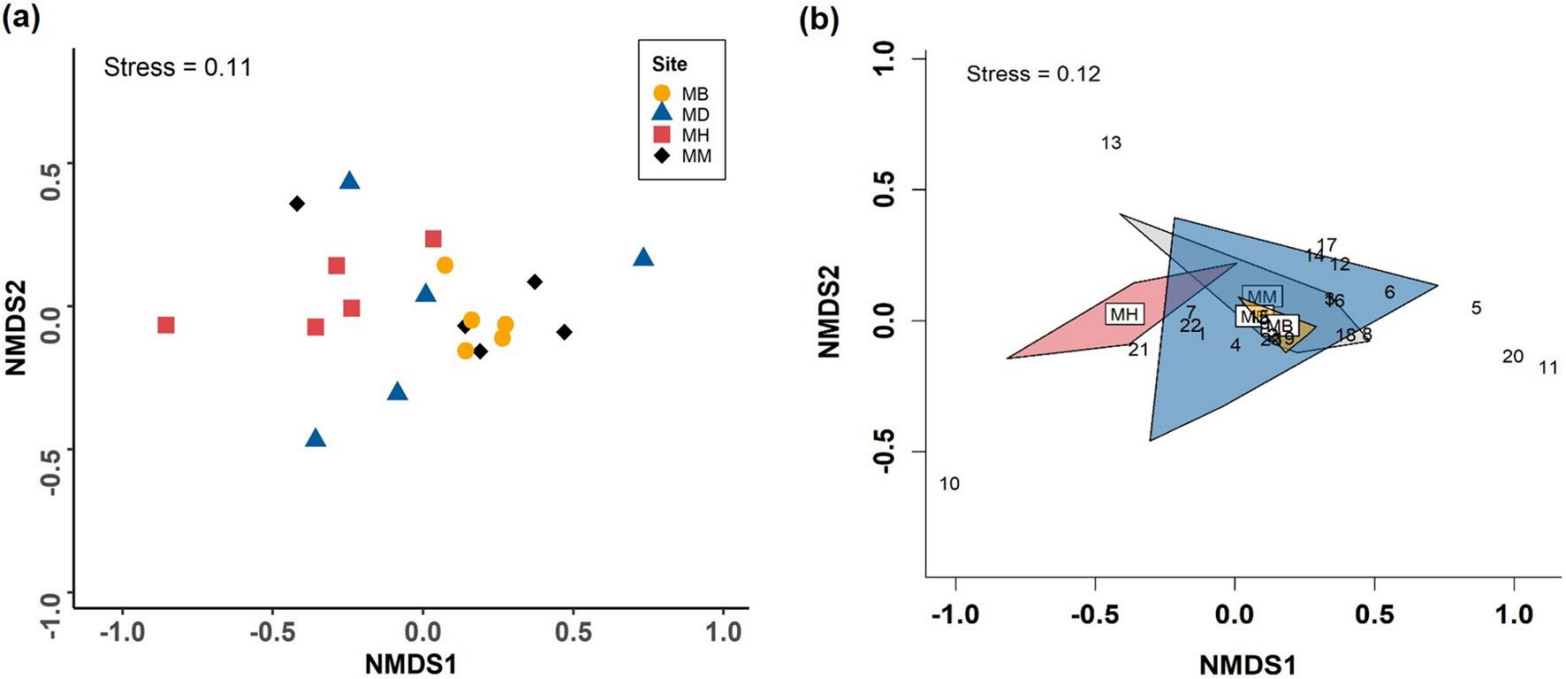
NMDS plots for VOCs emitted by mānuka at four different sites in winter. (**a**) Based on all the 34 VOCs identified from mānuka and (**b**) based on 23 VOCs with high contributions selected through SIMPER. The numbers on the graph represent the following compounds; (1) (*E*)-α-bergamotene, (2) (*E*)-β-caryophyllene, (3) (*Z*)-3-hexenal, (4) (*Z*)-3-hexenyl acetate, (5) (*Z*)-β-ocimene, (6) alloaromadendrene, (7) aromadendrene, (8) cadinadiene-1,4, (9) calamenene, (10) eucalyptol, (11) isoledene, (12) limonene, (13) o-cymene, (14) ylangene, (15) α-cubebene, (16) α-gurjunene, (17) α-pinene, (18) α-selinene, (19) β-chamigrene, (20) β-elemene, (21) β-myrcene, (22) β-pinene, (23) β-selinene. Mānuka – Broom (MB), Mānuka – *Dracophyllum* (MD), Mānuka – Heather (MH), Mānuka – Mānuka (MM).

The bouquet of volatiles produced by mānuka at the four sites also varied in winter (PERMANOVA; Pseudo-*F* = 2.05, *P* = 0.023, Fig. 3). The pairwise comparison revealed a significant variation between the conspecific stands and the site where mānuka and heather were the dominant species (Pseudo-*F* = 3.46, *P* = 0.023, Fig. 3). Again, VOCs varied significantly between the sites where mānuka occurs with the two invasive plants (Pseudo-*F* = 5.34, *P* = 0.005, Fig. 3). VOC composition did not vary between the mānuka – *Dracophyllum* site and the other three sites, although borderline significance was found for this site and the mānuka – heather site (Pseudo-*F* = 1.98,* P* = 0.050, Fig. 3, Supplementary Table S2). The similarity percentage analysis showed that 23 volatile compounds contributed to the variations detected in VOC emissions between the four sites in winter (Fig. 3b).

### Biotic and abiotic factors differ between sites

**Figure 4:**
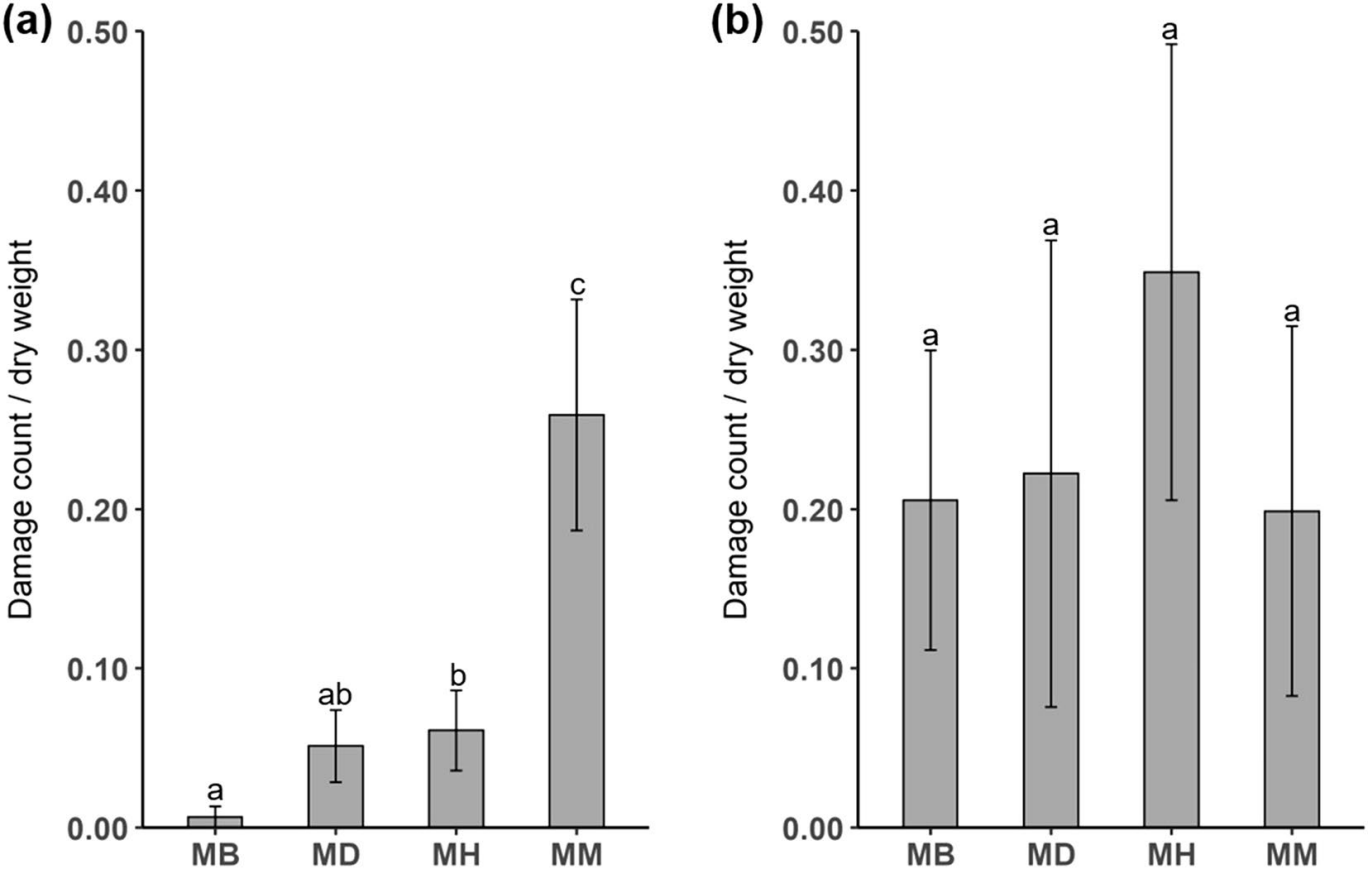
Herbivore damage on mānuka foliage expressed as mean ± SE damage counts/g in summer 2017 (**a**) and winter 2018 (**b**). N = 5 for all treatments at each site in both seasons. Different letters indicate a significant difference between sites. Mānuka – Broom (MB), Mānuka – *Dracophyllum* (MD), Mānuka – Heather (MH), and Mānuka – Mānuka (MM).

In summer, there was significantly higher herbivore damage on mānuka in the conspecific stands compared to the other sites (Kruskal–Wallis; *X*^2^ = 13.524, *df* = 3, *P* = 0.004, Fig. 4a). However, herbivore damage on mānuka did not differ between the four sites in winter (Kruskal–Wallis; *X*^2^ = 1.250, *df* = 3, *P* = 0.741, Fig. 4b).

**Figure 5:**
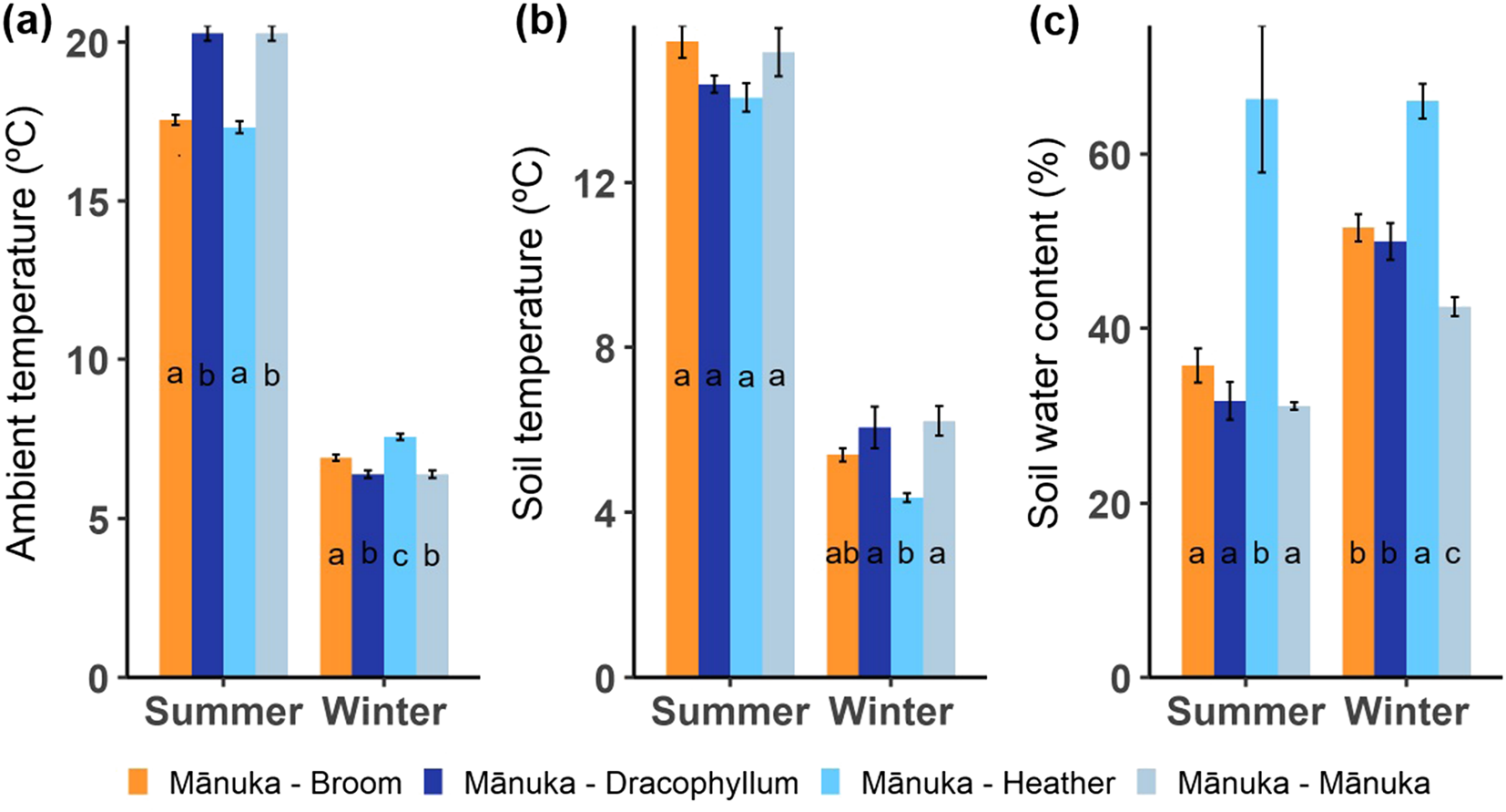
Comparison of (**a**) ambient temperature (**b**) soil temperature and (**c**) soil water content between study sites. Bars show mean ± SE of measured variables, and different letters indicate significant differences between sites.

There were significant differences in ambient daytime temperatures between the four sites (Fig. 5a) in both seasons (Kruskal–Wallis; *X*^2^ = 140.50, *df* = 3, *P* < 0.001 and *X*^2^ = 129.270, *df* = 3, *P* < 0.001 for summer and winter respectively).

Overall, soils from the four study sites had low levels of macro and micronutrients. Soil from broom- and heather-invaded sites had slightly higher levels of nitrogen, carbon and organic matter. Also, soil collected from the broom-invaded site was marginally higher in calcium and magnesium in both seasons (Table 1).

**Table 1:** Level of nutrients found in soils from study sites. 20 soil cores collected from each site were homogenised and used for the soil analysis of respective sites. Reference (medium range) represents Hills’ laboratories’ crop guides for mixed pasture. MB (Mānuka – Broom), MD (Mānuka – *Dracophyllum*), MH (Mānuka – Heather) and MM **(**Mānuka – Mānuka).

Soil properties	Summer 2017				Winter 2018				Reference (medium range)
	MB	MD	MH	MM	MB	MD	MH	MM	
Total nitrogen (%)	0.37	0.26	0.30	0.27	0.40	0.29	0.33	0.26	0.30–0.60
Total carbon (%)	4.70	3.40	6.00	3.40	5.50	4.80	6.50	4.00	NA
Phosphorus (me/100 g)	8.00	3.00	3.00	3.00	3.00	2.00	3.00	2.00	20–30
Sodium (me/100 g)	0.08	0.06	0.06	0.09	0.07	0.07	0.07	0.08	0.20–0.50
Magnesium (me/100 g)	0.72	0.54	0.31	0.56	0.80	0.72	0.42	0.77	1.00–1.60
Calcium (me/100 g)	3.00	2.20	1.60	2.20	3.70	3.00	2.00	3.10	4.0–10.0
Potassium (me/100 g)	0.24	0.21	0.24	0.23	0.25	0.26	0.32	0.28	0.40–0.60
Organic matter (%)	8.10	5.90	10.4	5.80	9.50	8.30	11.20	6.80	7.0–17.0
pH	6.00	6.00	5.70	6.00	5.80	5.80	5.70	6.00	5.80–6.20

me/100 g = Milliequivalents/100 g.

*NA* not applicable.

Soil temperature between the sites did not differ in summer (ANOVA; *F*_3,16_ = 2.462, *P* = 0.099) but was significantly different in winter (ANOVA; *F*_3,16_ = *7*.021, *P* = 0.003), with the mānuka – heather site having the lowest soil temperature (Fig. 5b).

Soil water content also differed significantly between the study sites in summer (Kruskal–Wallis; *X*^2^ = 12.440, *df* = 3, *P* = 0.006) and winter (ANOVA; *F*_3,16_ = 31.840, *P* < 0.001). The Mānuka – Heather site had significantly higher amounts of SWC than the other sites in both seasons (Fig. 5c).

### Influences of biotic and abiotic factors on volatile emissions

We investigated the effects of some environmental factors known to influence biogenic volatile organic compounds emission using PERMANOVA based on Euclidean distances. In all models, herbivory, ambient daytime temperature, nitrogen (as a proxy for soil nutrients) and soil water content (SWC) were used as predictors while the VOCs selected through SIMPER were response variables (Figs. 2b and 3b).

In summer, the emissions of 15 out of 27 VOCs were significantly affected by at least one of the tested environmental variables, with temperature, herbivory and nitrogen being major drivers. On the other hand, 12 volatile compounds (all terpenoids) were not affected by any of the tested variables (Supplementary Table S3). Differences in temperatures between sites significantly affected the emission of ten compounds (*Z*)-3-hexenol, cadinadiene-1,4, germacrene D, humulene, isoledene, α-selinene, β-selinene, 2-methyl-1-butanol acetate and 3-methyl-1-butanol acetate (Table 2). Herbivore damage was the second most important factor, having a significant effect on the emissions of eight compounds, (*Z*)-3-hexenol, copaene, germacrene D, α-cubebene, β-elemene, 2-methyl-1-butanol acetate and 3-methyl-1-butanol acetate (Table 2). Lastly, differences in soil nutrients (N) between sites had a significant effect on the emissions of four compounds, (*Z*)-3-hexenyl acetate, β-pinene, α-selinene and 2-heptanone (Table 2). No compounds were impacted by soil water content.

**Table 2:** Effects of environmental variables on the emission of VOCs selected through SIMPER for both summer and winter.

Compound	Environmental variables							
	Herbivory		Temperature		Nitrogen		SWC	
Summer 2017	*F*	*P*	*F*	*P*	*F*	*P*	*F*	*P*
(*Z*)-3-hexenol	5.140	**0.042**	10.102	**0.008**	0.040	0.840	0.410	0.487
(*Z*)-3-hexenyl acetate	3.810	0.076	1.360	0.259	5.540	**0.038**	0.020	0.898
β-pinene	0.130	0.730	1.608	0.231	7.892	**0.014**	0.209	0.575
Cadinadiene-1,4	2.923	0.089	4.333	**0.041**	0.737	0.410	0.007	0.915
Copaene	5.118	**0.043**	2.051	0.174	2.678	0.110	0.084	0.768
Germacrene D	11.302	**0.005**	14.951	**0.008**	1.449	0.247	0.0015	0.978
Humulene	3.526	0.061	5.648	**0.026**	0.498	0.487	0.389	0.520
Isoledene	0.509	0.431	6.160	**0.014**	0.383	0.552	0.007	0.924
α-cubebene	5.970	**0.023**	2.942	0.116	0.510	0.479	0.062	0.823
α-selinene	0.186	0.645	4.336	**0.048**	4.678	**0.041**	0.405	0.470
β-elemene	15.174	**0.006**	4.410	0.062	0.016	0.904	0.024	0.862
β-selinene	0.970	0.291	4.553	**0.042**	3.213	0.100	0.007	0.920
2-Heptanone	1.231	0.178	1.772	0.228	8.375	**0.014**	0.003	0.934
2-methyl-1-butanol acetate	22.426	**0.002**	6.321	**0.024**	0.572	0.469	1.150	0.262
3-methyl-1-butanol acetate	23.687	**0.001**	6.605	**0.015**	0.444	0.510	0.827	0.405
**Winter 2018**								
Aromadendrene	< 0.001	0.987	8.997	**0.011**	15.823	**0.003**	3.553	0.062
Cadinadiene-1,4	4.945	**0.045**	1.971	0.173	3.871	0.060	5.951	**0.036**
Calamenene	2.995	0.115	3.764	0.077	0.354	0.557	4.594	**0.045**
Isoledene	6.019	**0.036**	1.951	0.198	0.011	0.917	0.348	0.552
Alloaromadendrene	0.747	0.369	0.055	0.816	1.852	0.171	6.498	**0.025**
α-gurjunene	3.386	0.087	1.862	0.181	4.788	**0.045**	7.819	**0.021**
α-selinene	0.764	0.395	2.021	0.165	4.254	**0.048**	4.167	0.053
β-chamigrene	3.412	0.080	8.421	**0.011**	1.880	0.207	2.356	0.156
β-elemene	0.033	0.859	7.424	**0.015**	0.370	0.547	0.667	0.436
β-selinene	2.355	0.127	9.163	**0.010**	1.236	0.262	2.747	0.118

Pseudo-*F* (*F*) and *P* values (*P*) calculated using PERMANOVA based on Euclidean distances. Significant *P* values (*P* < 0.050) are highlighted in bold.

*SWC* soil water content.

In winter, differences in emissions of ten sesquiterpenes between the four study sites were explained by the tested environmental variables, while 13 compounds were not significantly affected by any of the predictor variables (Supplementary Table S3). Temperature differences between the sites affected the emission of aromadendrene, β-chamigrene, β-elemene and β-selinene (Table 2). The emission of cadinadiene-1,4, calamenene, alloaromadendrene and α-gurjunene were significantly affected by differences in soil water content between sites (Table 2). Similarly, differences in soil nutrients accounted for emissions of aromadendrene, α-gurjunene and α-selinene, while herbivore damage affected only the release of cadinadiene-1,4 and isoledene (Table 2).

## Discussion

Our study reveals that the New Zealand native plant mānuka is a rich producer of terpenoids, mostly sesquiterpenoids. Moreover, the study shows a natural seasonal and site-specific variation in the volatile emissions of this species. Emissions were often lower at sites where the exotic invasive weeds were present (Supplementary Table S4 and S5). The variable emission of most volatile compounds was explained by differences in air temperature, herbivory, soil nitrogen and soil water content levels between sites, but the observed effects were seasonal, being more pronounced during summer. This is consistent with previous reports showing effects of temperature, herbivory, soil nitrogen and soil water content on volatile emissions^7,12,36^.

Volatile emission had a clear seasonal pattern, mainly related to temperature. Elevated temperature is known to increase emissions of biogenic volatile organic compounds^7,37^. Temperature can directly affect VOC emissions by regulating the evaporation and release of compounds^38,39^. It can also control stomatal conductance, activities of enzymes and production of photosynthetic metabolites, which can all influence the emission of plant volatiles^40^. Among the environmental variables tested in our study, temperature differences between the sites affected the emission of several volatile compounds, including a green leaf volatile, terpenoids and other esters. Overall, temperatures were higher in the summer and emissions of all major VOCs groups increased at this time of the year. This observation supports the claim that current warming is likely to increase the global emissions of plant volatiles, which can affect their physiological and ecological functions^41^.

Herbivory is the most studied biotic factor concerning biogenic volatile emissions. In the field, plants are subjected to attack by numerous herbivores, and they have evolved a variety of defence mechanisms in response. Chemically, many stored volatile organic compounds are released by plants into the atmosphere when damaged by herbivores, but some compounds are also synthesised de novo when plants are under herbivore attack^42–44^. Herbivore loads and their impact vary with the season, as reflected by our data. During summer we observed the most damage to foliage on mānuka in the conspecific stands, and this was probably caused by the high numbers of mānuka beetles (*Pyronota festiva*). At other sites where mānuka occurs with the native species *Dracophyllum* there was less damage despite mānuka beetle numbers being high (Supplementary Fig. S1). We did not investigate the factors accounting for the lower damage at this site. However, possible reasons could include higher defence responses such as the increased emissions of some monoterpenoids by mānuka at this site (Supplementary Table S4 and S5) or relatively recent migration of the beetles to this site at the time of the experiment. In contrast, at sites where mānuka occurs with broom and heather, both damage levels and mānuka beetle numbers were low (Fig. 4, Supplementary Fig. S1). This may indicate a disruption in communication between mānuka and its principal herbivore and suggests that the abundance of herbivorous arthropod is reduced at sites where invasive plants are dominant^16,45^.

In summer, herbivory on mānuka accounted for the emissions of (*Z*)-3-hexenol, copaene, germacrene D, α-cubebene, β-elemene and some esters. These herbivore-induced volatiles have been identified as key elements of plant defence against herbivores^3,46–48^. In contrast, the effect of herbivore damage on VOC emissions was almost negligible in winter when herbivores were mostly absent. This suggests that in summer, when there is a higher threat of herbivory, plants could benefit by emitting higher amounts of VOCs to directly repel herbivores and attract their natural enemies^49–52^. Due to the small number of arthropods identified at all sites in winter (data not shown), it is possible high foliage damage recorded in this season is cumulative and occurred in preceding seasons. Other factors, like cold-stress, may also impact VOC emissions in winter, and we recommend more studies to investigate plant volatile emissions in cold environments.

Invasive woody species are known to impact the microclimate of the sites they invade, reducing the direct effects of high radiation and temperature, increasing water availability and causing accumulation of nutrients and organic matter in the soil^53,54^. For example, soil from heather stands in the Tongariro National Park in New Zealand was extremely acidic and had a high level of carbon and nitrogen compared with soils from native stands^55^. Several studies assessing the impact of broom invasion on soil properties have also reported increased levels of other organic matter and C stores, N and P at invaded sites^56–58^. This is consistent with our results, where we found lower ambient temperatures in the invaded sites during summer, higher water availability in the sites invaded by heather, and higher carbon, nitrogen, and organic matter contents in both heather- and broom-invaded sites. Among soil nutrients, N is the most studied in relation to VOC emissions, and effects are plant species and compound dependent^7,59^. We found a significant effect of N on the emission of some VOCs in both summer and winter, suggesting potential changes in the plant’s biochemistry resulting from modification in soil composition by the invasive species. To improve our knowledge of the relationship between exotic weeds and soil chemistry of the new habitat, we suggest further studies to test whether differences in soil properties often reported during plant invasions are caused by the presence of exotic weeds rather than the invaders’ preference to grow at local nutrient sites.

Our results also show an effect of differences in soil water content between the sites on emission of the sesquiterpenes cadinadiene-1,4, calamenene, alloaromadendrene and α-gurjunene. This effect of soil water content on VOC emissions was not detected in summer, which suggests that under natural conditions plants may give priority to certain stressors such as higher temperature and herbivory in summer. In addition, the effect of water availability on plant volatile emissions may vary depending on the severity and duration of the stress, with opposing results in the literature^36,40^.

Previous studies have reported variation in volatile emissions between conspecific and heterospecific stands. For instance, *Pinus halepensis* reduced its VOC emission when sharing a pot with *Quercus ilex* compared with conspecifics^60^. In the field, the Mediterranean plant *Rosmarinus officinalis* also reduced its emission of monoterpenes when neighbored with *Pinus halepensis*^61^. A recent study also showed that the invasive plant *Calluna vulgaris* produces lower levels of volatiles at a site where it co-exists with another invasive plant *Cytisus scoparius*, which is a nitrogen-fixer, capable of modifying soil properties^16^. However, other studies also show lower emissions when plants were paired with conspecifics^62^. In the present study, we found that VOC emissions by mānuka were often lower at sites where mānuka co-occurred with heterospecifics, particularly the two invasive plants (Supplementary Table S4 and S5).

Considering the limitation of small sample size on the present study, we recommend further studies to investigate whether reduction in VOC emissions by plants in heterospecific stands is widespread and whether the co-evolutionary history between neighbouring plants influences emissions. Such studies should include more study sites and perform experiments for much longer periods, covering different developmental stages, age of target plants and changes in other conditions in study sites. Another important aspect to consider in future studies is the genetic relatedness of plants in conspecific and heterospecific stands since VOC emissions and their impact can vary between close and distance relatives^63,64^. In the present study, it is possible that mānuka plants at a site are more closely related thereby producing unique volatile blend compared with those at other sites, which may^65^ or may not^64^ affect their ecological roles. Therefore, we recommend future studies to perform detailed genetic analysis of plants to ensure that changes in VOC emissions are not merely reflecting the genetic variability of tested plants.

## Conclusion

We have, for the first time, characterised the volatile emissions of mānuka plants and provided evidence for natural variation in VOC emissions. Our results show that variability in the emission of most compounds produced by mānuka is influenced by microclimatic factors and herbivory, with strong seasonal differences. Our study shows that temperature is a significant factor influencing VOC emissions, suggesting that current warming is likely to increase the global emissions of plant volatiles and affect their ecological roles. Although different biotic and abiotic factors explained emissions of most compounds, there were also VOC emissions that were not explained by these variables, yet their relative proportions varied between the sites (Supplementary Table S4 and S5). Therefore, further studies are needed to investigate other factors that may influence VOC emissions such as genetic variation in plants, plant community effects, belowground herbivory or association with beneficial microbes or pathogens.

## Supplementary information

Supplementary information

## Data Availability

All relevant datasets are available upon request.
