# Supplementary material for: Seasonal and environmental variation in volatile emissions of the New Zealand native plant Leptospermum scoparium in weed-invaded and non-invaded sites
Source: Sci Rep. 2020 Jul 16;10:11736. doi: 10.1038/s41598-020-68386-4 (PMC7366711; doi:10.1038/s41598-020-68386-4)
Supplement: Supplementary file 1 — Supplementary information [file 41598_2020_68386_MOESM1_ESM.docx]

**Seasonal and environmental variation in volatile emissions of the New Zealand native plant *Leptospermum scoparium* in weed-invaded and non-invaded sites**

Evans Effah^1^, D. Paul Barrett^1^, Paul G. Peterson^2^, Murray A. Potter^1^, Jarmo K. Holopainen^3^, *Andrea Clavijo McCormick^1^

**Table S1**. GPS coordinates for experimental sites

| **Site / dominant woody species** | **Location (GPS coordinates)** | **Sampled area** |
| --- | --- | --- |
| Mānuka - Broom | Long. 175.6685 – Lat. -39.451283 | 23 m × 11 m |
| Mānuka - *Dracophyllum* | Long. 175.685483 – Lat. -39.432933 | 24 m × 12 m |
| Mānuka - Heather | Long. 175.734317 – Lat. -39.314683 | 24 m × 13 m |
| Mānuka - Mānuka | Long. 175.685483 – Lat. -39.432933 | 27 m × 14 m |

**Table S2.** Pairwise comparison of volatile profile of mānuka between sites. Bold fonts show significant difference

| **Pairs** | **DF** | **Sum of Squares** | **Pseudo-*F*** | **R^2^** | ***P*-value** |
| --- | --- | --- | --- | --- | --- |
| **Summer** |  |  |  |  |  |
| **MM vs. MH** | **1** | **0.372** | **7.399** | **0.481** | **0.012** |
| MM vs. MD | 1 | 0.108 | 1.610 | 0.167 | 0.176 |
| **MM vs. MB** | **1** | **0.305** | **7.494** | **0.484** | **0.006** |
| MH vs. MD | 1 | 0.177 | 2.420 | 0.232 | 0.080 |
| **MH vs. MB** | **1** | **0.169** | **3.623** | **0.312** | **0.009** |
| MD vs. MB | 1 | 0.137 | 2.147 | 0.212 | 0.113 |
|  |  |  |  |  |  |
| **Winter** |  |  |  |  |  |
| **MM vs. MH** | **1** | **0.258** | **3.557** | **0.308** | **0.035** |
| MM vs. MD | 1 | 0.065 | 0.650 | 0.075 | 0.679 |
| MM vs. MB | 1 | 0.042 | 0.777 | 0.088 | 0.692 |
| MH vs. MD | 1 | 0.186 | 1.983 | 0.199 | 0.050 |
| **MH vs. MB** | **1** | **0.269** | **5.540** | **0.409** | **0.012** |
| MD vs. MB | 1 | 0.099 | 1.317 | 0.141 | 0.234 |

**Abbreviations:** Versus (vs.), Mānuka – Mānuka (MM), Mānuka – Heather (MH), Mānuka – *Dracophyllum* (MD), Mānuka – Broom (MB)

**Table S3.** Effects of environmental variables on emissions of VOCs selected through SIMPER for both summer and winter. *F*-values (Pseudo-F) and *p*-values (*P*) calculated using PERMANOVA based on Euclidean distances. Table shows VOCs whose emissions were not significantly affected by any of the tested variables.

| **Compound** | **Environmental variables** | | | | | | | |
| --- | --- | --- | --- | --- | --- | --- | --- | --- |
|  | **Herbivory** | | **Temperature** | | **Nitrogen** | | **SWC** | |
| **Summer 2017** | ***F*** | ***P*** | ***F*** | ***P*** | ***F*** | ***P*** | ***F*** | ***P*** |
| (*Z*)-β-ocimene | 0.118 | 0.691 | 0.066 | 0.803 | 0.379 | 0.527 | 0.431 | 0.418 |
| Lemonol | 4.244 | 0.061 | 0.286 | 0.597 | 2.978 | 0.104 | 0.095 | 0.730 |
| Linalool | 1.054 | 0.223 | 2.574 | 0.115 | 0.808 | 0.408 | 0.041 | 0.806 |
| α-pinene | 1.557 | 0.206 | 0.379 | 0.536 | 2.290 | 0.145 | 0.324 | 0.599 |
| o-cymene | 2.996 | 0.089 | 4.442 | 0.057 | <0.001 | 0.985 | 0.219 | 0.672 |
| β-myrcene | 0.230 | 0.643 | 0.005 | 0.935 | 2.081 | 0.155 | 1.170 | 0.330 |
| (*E*)-β-caryophyllene | 3.711 | 0.080 | 4.417 | 0.058 | 0.403 | 0.543 | 0.264 | 0.583 |
| Aromadendrene | 0.011 | 0.911 | 0.471 | 0.503 | 2.387 | 0.141 | 0.051 | 0.813 |
| Calamenene | 3.654 | 0.083 | 2.993 | 0.105 | 1.217 | 0.297 | <0.001 | 0.997 |
| (*Z*,*E*)-α-farnesene | 2.456 | 0.093 | 0.488 | 0.530 | 0.001 | 0.977 | 0.027 | 0.814 |
| (*E*,*E*)-α-farnesene | 2.453 | 0.082 | 0.683 | 0.424 | 0.002 | 0.970 | 0.012 | 0.902 |
| β-chamigrene | 0.025 | 0.863 | 0.712 | 0.420 | 0.797 | 0.393 | 0.001 | 0.972 |
| **Winter 2018** |  |  |  |  |  |  |  |  |
| (*Z*)-3-hexenyl acetate | 0.381 | 0.554 | 0.790 | 0.381 | 0.160 | 0.705 | 0.189 | 0.676 |
| (*Z*)-3-hexenal | 0.381 | 0.538 | 0.656 | 0.414 | 0.010 | 0.927 | 0.019 | 0.893 |
| (*Z*)-β-ocimene | 0.117 | 0.757 | 0.001 | 0.981 | 0.043 | 0.853 | 0.001 | 0.980 |
| Limonene | 1.327 | 0.242 | 2.000 | 0.189 | 0.028 | 0.862 | 1.805 | 0.158 |
| o-cymene | 0.195 | 0.667 | 1.369 | 0.271 | 0.624 | 0.467 | 0.680 | 0.421 |
| α-pinene | 0.053 | 0.812 | 1.547 | 0.250 | 0.088 | 0.800 | 0.006 | 0.937 |
| β-myrcene | 0.030 | 0.844 | 0.774 | 0.369 | 0.190 | 0.656 | 0.347 | 0.528 |
| β-pinene | 0.358 | 0.571 | 0.021 | 0.881 | 4.148 | 0.054 | 1.702 | 0.202 |
| Eucalyptol | 4.952 | 0.055 | 2.033 | 0.171 | <0.001 | 0.997 | 0.025 | 0.869 |
| (*E*)-α-bergamotene | 0.944 | 0.339 | 0.498 | 0.506 | 0.070 | 0.801 | 1.681 | 0.203 |
| (*E*)-β-caryophyllene | 2.946 | 0.113 | 2.360 | 0.153 | 0.118 | 0.763 | 0.475 | 0.469 |
| Ylangene | 0.591 | 0.436 | 0.062 | 0.823 | <0.001 | 0.997 | 0.103 | 0.751 |
| α-cubebene | 2.350 | 0.146 | 3.399 | 0.063 | 0.062 | 0.832 | 1.696 | 0.202 |

SWC = soil water content

**Table S4.** Emission rates of VOCs identified from the headspace of mānuka at four different sites in summer. Comparison performed using generalized linear model assuming Gamma distribution (log-link) with VOCs as response and sites and predictor variables. Likelihood ratio test used to test the significance of predictor. Prior to modeling, a small constant (0.0001) was added to all response variables. P-values in bold font indicates significant difference (P < 0.05). N = 5 replicates for each site.

|  | **Mean ± SE emission rate per site (ng gDW^-1^h^-1^)** | | | | **Likelihood ratio test** | | |
| --- | --- | --- | --- | --- | --- | --- | --- |
| **Compound** | **MM** | **MH** | **MD** | **MB** | ***X*^2^** | **DF** | ***P*-value** |
| **Green leaf volatiles**  **(GLVs)** | |  |  |  |  |  |  |
| (*E*)-2-hexenal | 0.08 ± 0.05 | ND | 0.05 ± 0.04 | ND | 28.97 | 3 | **< 0.001** |
| (*Z*)-3-hexenol | 0.90 ± 0.24 | 0.14 ± 0.04 | 0.77 ± 0.37 | 0.18 ± 0.05 | 18.08 | 3 | **< 0.001** |
| (*Z*)-3-hexenyl acetate^i^ | 10.23 ± 2.00 | 2.13 ± 0.54 | 3.39 ± 0.96 | 5.56 ± 1.02 | 16.55 | 3 | **0.001** |
| Hexanol | 0.02 ± 0.01 | 0.01 ± 0.01 | 0.02 ± 0.02 | 0.02 ± 0.02 | 0.58 | 3 | 0.900 |
| Hexyl acetate | 0.24 ± 0.06 | 0.04 ± 0.02 | 0.11 ± 0.02 | 0.14 ± 0.06 | 5.17 | 3 | 0.160 |
| **Total GLVs** | 11.47 ± 2.22 | 2.33 ± 0.58 | 4.34 ± 1.00 | 5.80 ± 1.09 | 17.17 | 3 | **< 0.001** |
|  |  |  |  |  |  |  |  |
| **Monoterpenoids**  **(MTs)** | |  |  |  |  |  |  |
| (*E*)- β-ocimene | 0.05 ± 0.01 | 0.06 ± 0.03 | 0.02 ± 0.02 | ND | 18.81 | 3 | **<0.001** |
| (*Z*)-β-ocimene | 0.57 ± 0.45 | 0.54 ± 0.42 | 0.29 ± 0.15 | 0.17 ± 0.04 | 3.13 | 3 | 0.371 |
| Lemonol | 0.35 ± 0.14 | 0.26 ± 0.08 | 0.40 ± 0.25 | 0.08 ± 0.05 | 2.91 | 3 | 0.406 |
| Limonene^i^ | 0.16 ± 0.04 | 0.05 ± 0.02 | 0.13 ± 0.05 | 0.12 ± 0.04 | 3.54 | 3 | 0.316 |
| Linalool^i^ | 2.77 ± 1.26 | 0.98 ± 0.27 | 6.22 ± 4.92 | 0.18 ± 0.05 | 18.72 | 3 | **< 0.001** |
| Terpinen-4-ol | 0.02 ± 0.01 | ND | 0.03 ± 0.02 | ND | 28.76 | 3 | **< 0.001** |
| α-phellandrene | 0.03 ± 0.02 | ND | 0.03 ± 0.02 | 0.01 ± 0.01 | 14.33 | 3 | **0.002** |
| α-pinene^i^ | 0.29 ± 0.16 | 0.26 ± 0.14 | 1.93 ± 1.15 | 1.46 ± 0.27 | 7.75 | 3 | 0.052 |
| o-cymene | 0.36 ± 0.13 | 0.04 ± 0.02 | 0.35 ± 0.24 | 0.05 ± 0.04 | 5.43 | 3 | 0.143 |
| β-myrcene | 0.78 ± 0.44 | 0.51 ± 0.30 | 0.53 ± 0.16 | 1.12 ± 0.39 | 1.47 | 3 | 0.690 |
| β-pinene^i^ | 0.64 ± 0.22 | 0.35 ± 0.07 | 0.31 ± 0.04 | 1.67 ± 0.70 | 16.89 | 3 | **0.001** |
| Eucalyptol^i^ | 0.15 ± 0.09 | 0.01 ± 0.01 | 0.08 ± 0.06 | 0.04 ± 0.02 | 4.07 | 3 | 0.254 |
| **Total MTs** | 6.17 ± 1.84 | 3.06 ± 1.04 | 10.33 ± 6.73 | 4.88 ± 1.31 | 5.59 | 3 | 0.133 |
|  |  |  |  |  |  |  |  |
| **Sesquiterpenoids**  **(SQTs)** | |  |  |  |  |  |  |
| (*E*)-α-bergamotene | 0.20 ± 0.08 | 0.09 ± 0.03 | 0.10 ± 0.03 | 0.10 ± 0.04 | 0.99 | 3 | 0.803 |
| (*E*)-β-caryophyllene^i^ | 13.89 ± 8.27 | 2.11 ± 0.57 | 10.87 ± 5.10 | 0.71 ± 0.24 | 19.38 | 3 | **< 0.001** |
| Alloaromadendrene | 0.14 ± 0.02 | 0.07 ± 0.02 | 0.13 ± 0.08 | 0.19 ± 0.08 | 1.60 | 3 | 0.659 |
| Aromadendrene | 0.15 ± 0.06 | 0.07 ± 0.01 | 0.14 ± 0.13 | 0.33 ± 0.19 | 3.21 | 3 | 0.360 |
| Cadinadiene-1,4 | 0.68 ± 0.18 | 0.09 ± 0.03 | 0.85 ± 0.54 | 0.26 ± 0.11 | 9.82 | 3 | **0.020** |
| Calamenene | 2.62 ± 0.49 | 0.48 ± 0.12 | 2.40 ± 1.41 | 1.31 ± 0.54 | 10.20 | 3 | **0.017** |
| Caryophyllene oxide | 0.24 ± 0.09 | 0.02 ± 0.01 | 0.10 ± 0.06 | 0.03 ± 0.02 | 6.14 | 3 | 0.105 |
| Copaene | 0.53 ± 0.12 | 0.06 ± 0.03 | 0.38 ± 0.24 | 0.25 ± 0.10 | 5.07 | 3 | 0.167 |
| Germacrene D | 0.27 ± 0.05 | ND | 0.20 ± 0.12 | 0.04 ± 0.03 | 27.76 | 3 | **< 0.001** |
| Ylangene | 0.12 ± 0.05 | 0.03 ± 0.01 | 0.13 ± 0.08 | 0.06 ± 0.02 | 3.35 | 3 | 0.341 |
| α-bisabolol | 0.05 ± 0.01 | ND | 0.04 ± 0.03 | ND | 36.62 | 3 | **< 0.001** |
| α-cubebene | 1.09 ± 0.27 | 0.21 ± 0.04 | 0.88 ± 0.47 | 0.40 ± 0.16 | 10.29 | 3 | **0.016** |
| (*E*,*E*)-α-farnesene | 2.99 ± 1.61 | 0.14 ± 0.14 | ND | ND | 33.79 | 3 | **< 0.001** |
| α-gurjunene | 0.13 ± 0.03 | 0.03 ± 0.01 | 0.18 ± 0.10 | 0.15 ± 0.07 | 5.75 | 3 | 0.125 |
| Humulene^i^ | 0.75 ± 0.42 | 0.12 ± 0.03 | 0.67 ± 0.27 | 0.05 ± 0.01 | 14.92 | 3 | **0.001** |
| Isoledene | 0.73 ± 0.30 | 0.05 ± 0.02 | 1.36 ± 0.90 | 0.24 ± 0.10 | 14.59 | 3 | **0.002** |
| α-selinene | 0.22 ± 0.06 | 0.02 ± 0.01 | 0.38 ± 0.27 | 0.23 ± 0.09 | 10.27 | 3 | **0.016** |
| β-chamigrene | 1.16 ± 0.73 | 0.43 ± 0.18 | 4.16 ± 2.65 | 1.89 ± 0.64 | 4.10 | 3 | 0.250 |
| β-elemene | 4.06 ± 1.51 | 0.66 ± 0.21 | 3.19 ± 1.83 | 0.56 ± 0.16 | 16.72 | 3 | **0.001** |
| (*E*)-β-farnesene | 0.06 ± 0.03 | 0.03 ± 0.03 | 0.03 ± 0.03 | ND | 26.57 | 3 | **< 0.001** |
| (*Z*,*E*)-α-farnesene | 0.53 ± 0.32 | 0.04 ± 0.04 | ND | ND | 32.50 | 3 | **< 0.001** |
| β-selinene | 1.84 ± 0.27 | 0.26 ± 0.10 | 2.84 ± 1.73 | 1.52 ± 0.48 | 9.90 | 3 | **0.019** |
| γ-elemene | 0.19 ± 0.10 | 0.01 ± 0.01 | 0.17 ± 0.10 | 0.01 ± 0.01 | 8.96 | 3 | **0.030** |
| δ-cadinene | 0.10 ± 0.06 | ND | 0.18 ± 0.13 | ND | 30.13 | 3 | **< 0.001** |
| **Total SQTs** | 32.70 ±12.09 | 4.99 ± 1.27 | 29.38 ± 15.59 | 8.32 ± 2.94 | 14.23 | 3 | **0.003** |
|  |  |  |  |  |  |  |  |
| **Aldehydes** |  |  |  |  |  |  |  |
| Decanal | 0.07 ± 0.01 | 0.12 ± 0.04 | 0.06 ± 0.01 | 0.15 ± 0.03 | 11.30 | 3 | **0.010** |
| Heptanal | 0.04 ± 0.03 | 0.03 ± 0.03 | 0.04 ± 0.02 | 0.06 ± 0.04 | 0.32 | 3 | 0.957 |
| Nonanal | 0.11 ± 0.02 | 0.23 ± 0.09 | 0.10 ± 0.04 | 0.32 ± 0.06 | 5.16 | 3 | 0.161 |
| Octanal | 0.04 ± 0.01 | 0.06 ± 0.02 | 0.04 ± 0.01 | 0.08 ± 0.02 | 1.51 | 3 | 0.681 |
| **Total aldehydes** | 0.26 ± 0.03 | 0.43 ± 0.16 | 0.24 ± 0.06 | 0.61 ± 0.11 | 8.19 | 3 | **0.042** |
|  |  |  |  |  |  |  |  |
| **Other esters** |  |  |  |  |  |  |  |
| 3-methyl-1-butanol acetate | 0.39 ± 0.12 | 0.04 ± 0.03 | 0.08 ± 0.03 | ND | 27.11 | 3 | **< 0.001** |
| 2-methyl-1-butanol acetate | 0.26 ± 0.09 | 0.03 ± 0.02 | 0.06 ± 0.02 | ND | 26.55 | 3 | **< 0.001** |
| (*Z)*-3-octenyl acetate | 0.03 ± 0.01 | ND | 0.04 ± 0.04 | 0.01 ± 0.01 | 12.69 | 3 | **0.005** |
| Hexyl 2-methylbutyrate | 0.14 ± 0.04 | 0.23 ± 0.02 | 0.13 ± 0.03 | 0.23 ± 0.01 | 1.4319 | 3 | 0.698 |
| β-phenethyl acetate | 0.05 ± 0.02 | ND | ND | ND | 101.32 | 3 | **< 0.001** |
| **Total esters** | 0.73 ± 0.22 | 0.07 ± 0.05 | 0.18 ± 0.07 | 0.01 ± 0.01 | 11.324 | 3 | **0.010** |
|  |  |  |  |  |  |  |  |
| **Ketones** |  |  |  |  |  |  |  |
| 2-heptanone | 0.01 ± 0.01 | ND | ND | 0.62 ± 0.40 | 38.33 | 3 | **< 0.001** |
|  |  |  |  |  |  |  |  |
| **Total VOC emissions** | 51.34 ±14.81 | 10.89 ± 2.74 | 44.47 ± 22.49 | 20.24 ± 4.85 | 12.50 | 3 | **0.006** |

**Abbreviations:** Mānuka – Broom (MB), Mānuka – *Dracophyllum* (MD), Mānuka – Heather (MH), and Mānuka – Mānuka (MM).

^i^ Compounds verified by authentic standards

**Table S5**. Emission rates of VOCs identified from the headspace of mānuka at four different sites in winter. Comparison performed using generalized linear model assuming Gamma distribution (log-link) with VOCs as response and sites and predictor variables. Likelihood ratio test used to test the significance of predictor. Prior to modeling, a small constant (0.0001) was added to all response variables. P-values in bold font indicates significant difference (P < 0.05). N = 5 replicates for each site.

|  | **Mean ± SE emission rate per site (ng gDW^-1^h^-1^)** | | | | **Likelihood ratio test** | | |
| --- | --- | --- | --- | --- | --- | --- | --- |
| **Compound** | **MM** | **MH** | **MD** | **MB** | ***X*^2^** | **DF** | ***P*-value** |
| **Green leaf volatiles**  **(GLVs)** | |  |  |  |  |  |  |
| (*Z*)-3-hexenal | 0.09 ± 0.06 | 0.02 ± 0.01 | 0.05 ± 0.04 | 0.05 ± 0.05 | 1.13 | 3 | 0.770 |
| (*Z*)-3-hexenyl acetate^i^ | 0.27 ± 0.13 | 0.11 ± 0.03 | 0.26 ± 0.14 | 0.20 ± 0.02 | 2.37 | 3 | 0.499 |
| (*Z*)-3-hexenol | 0.04 ± 0.02 | ND | 0.02 ± 0.02 | 0.05 ± 0.02 | 14.05 | 3 | **0.003** |
| **Total GLVs** | 0.41 ± 0.19 | 0.13 ± 0.03 | 0.34 ± 0.20 | 0.29 ± 0.04 | 2.96 | 3 | 0.398 |
|  |  |  |  |  |  |  |  |
| **Monoterpenoids**  **(MTs)** | |  |  |  |  |  |  |
| (*Z*)-β-ocimene | 0.10 ± 0.07 | 0.06 ± 0.04 | 0.09 ± 0.09 | 0.06 ± 0.04 | 0.21 | 3 | 0.976 |
| Limonene^i^ | 0.09 ± 0.02 | 0.04 ± 0.02 | 0.28 ± 0.21 | 0.07 ± 0.02 | 5.11 | 3 | 0.164 |
| Linalool^i^ | 0.03 ± 0.02 | 0.03 ± 0.02 | 0.06 ± 0.05 | 0.02 ± 0.02 | 1.14 | 3 | 0.769 |
| α-pinene^i^ | 0.60 ± 0.21 | 0.24 ± 0.07 | 5.66 ± 4.36 | 0.85 ± 0.34 | 14.46 | 3 | **0.002** |
| β-myrcene | 0.51 ± 0.32 | 1.20 ± 0.92 | 0.37 ± 0.17 | 0.79 ± 0.31 | 1.28 | 3 | 0.735 |
| β-pinene^i^ | 0.49 ± 0.21 | 0.46 ± 0.15 | 0.75 ± 0.30 | 1.03 ± 0.28 | 3.67 | 3 | 0.299 |
| γ-terpinene | 0.04 ± 0.04 | 0.05 ± 0.05 | 0.13 ± 0.07 | 0.03 ± 0.03 | 1.50 | 3 | 0.682 |
| o-cymene | 0.19 ± 0.13 | 0.08 ± 0.05 | 0.30 ± 0.13 | 0.04 ± 0.04 | 2.07 | 3 | 0.559 |
| Eucalyptol^i^ | 0.02 ± 0.02 | 0.03 ± 0.03 | 0.84 ± 0.80 | 0.01 ± 0.01 | 9.76 | 3 | **0.020** |
| **Total MTs** | 2.06 ± 0.25 | 2.18 ± 1.19 | 8.47 ± 5.11 | 2.89 ± 0.86 | 8.71 | 3 | **0.033** |
|  |  |  |  |  |  |  |  |
| **Sesquiterpenoids**  **(SQTs)** | |  |  |  |  |  |  |
| (*E*)-α-bergamotene | 0.12 ± 0.04 | 0.08 ± 0.03 | 0.22 ± 0.12 | 0.12 ± 0.03 | 1.19 | 3 | 0.755 |
| (*E*)-β-caryophyllene^i^ | 0.67 ± 0.42 | 0.11 ± 0.03 | 0.43 ± 0.23 | 0.38 ± 0.08 | 9.81 | 3 | **0.020** |
| Alloaromadendrene | 0.08 ± 0.04 | 0.05 ± 0.03 | 0.07 ± 0.06 | 0.14 ± 0.04 | 0.73 | 3 | 0.866 |
| Aromadendrene | 0.04 ± 0.03 | 0.11 ± 0.03 | 0.05 ± 0.04 | 0.29 ± 0.05 | 4.59 | 3 | 0.204 |
| Cadinadiene-1,4 | 0.23 ± 0.08 | 0.04 ± 0.01 | 0.16 ± 0.10 | 0.26 ± 0.05 | 5.08 | 3 | 0.166 |
| Calamenene | 1.51 ± 0.37 | 0.37 ± 0.11 | 1.08 ± 0.66 | 1.12 ± 0.23 | 7.96 | 3 | **0.047** |
| Caryophyllene oxide | 0.07 ± 0.04 | ND | 0.02 ± 0.01 | ND | 27.38 | 3 | **< 0.001** |
| Copaene | 0.26 ± 0.07 | 0.08 ± 0.02 | 0.23 ± 0.15 | 0.21 ± 0.05 | 6.16 | 3 | 0.104 |
| α-selinene | 0.38 ± 0.14 | 0.05 ± 0.01 | 0.32 ± 0.19 | 0.53 ± 0.15 | 6.93 | 3 | 0.074 |
| β-chamigrene | 2.06 ± 0.51 | 0.26 ± 0.03 | 1.79 ± 0.98 | 1.71 ± 0.19 | 18.66 | 3 | **< 0.001** |
| β-elemene | 0.14 ± 0.05 | ND | 0.20 ± 0.12 | 0.08 ± 0.02 | 21.75 | 3 | **< 0.001** |
| Humulene^i^ | 0.03 ± 0.03 | ND | 0.05 ± 0.04 | ND | 26.08 | 3 | **< 0.001** |
| Isoledene | 0.17 ± 0.10 | 0.01 ± 0.01 | 0.16 ± 0.13 | 0.10 ± 0.05 | 3.6 | 3 | 0.308 |
| Ylangene | 0.06 ± 0.02 | 0.06 ± 0.02 | 0.25 ± 0.22 | 0.10 ± 0.04 | 2.79 | 3 | 0.424 |
| α-amorphene | 0.05 ± 0.04 | 0.04 ± 0.03 | 0.04 ± 0.04 | 0.07 ± 0.06 | 0.16 | 3 | 0.984 |
| α-cubebene | 0.46 ± 0.11 | 0.13 ± 0.05 | 0.45 ± 0.30 | 0.26 ± 0.07 | 7.21 | 3 | 0.066 |
| α-gurjunene | 0.11 ± 0.03 | 0.04 ± 0.02 | 0.12 ± 0.08 | 0.16 ± 0.02 | 2.59 | 3 | 0.459 |
| Germacrene D | 0.05 ± 0.02 | 0.01 ± 0.01 | 0.02 ± 0.02 | 0.03 ± 0.02 | 1.62 | 3 | 0.655 |
| β-selinene | 2.42 ± 0.63 | 0.30 ± 0.01 | 1.92 ± 0.98 | 1.77 ± 0.19 | 18.93 | 3 | **< 0.001** |
| **Total SQTs** | 8.91 ± 2.28 | 1.76 ± 0.39 | 7.58 ± 4.39 | 7.32 ± 0.93 | 12.56 | 3 | **0.006** |
|  |  |  |  |  |  |  |  |
| **Aldehydes** |  |  |  |  |  |  |  |
| Decanal | 0.03 ± 0.02 | 0.03 ± 0.01 | 0.04 ± 0.02 | 0.01 ± 0.01 | 1.37 | 3 | 0.713 |
| Heptanal | 0.02 ± 0.02 | 0.01 ± 0.01 | 0.01 ± 0.01 | 0.03 ± 0.02 | 2.20 | 3 | 0.532 |
| Nonanal | 0.07 ± 0.02 | 0.08 ± 0.03 | 0.04 ± 0.03 | 0.09 ± 0.01 | 0.68 | 3 | 0.879 |
| **Total aldehydes** | 0.12 ± 0.03 | 0.11 ± 0.03 | 0.10 ± 0.04 | 0.13 ± 0.02 | 0.14 | 3 | 0.986 |
|  |  |  |  |  |  |  |  |
| **Total VOC emissions** | 11.49 ± 2.42 | 4.18 ± 1.57 | 16.48 ± 9.63 | 10.63 ±1.17 | 8.58 | 3 | 0.035 |

**Abbreviations:** Mānuka – Broom (MB), Mānuka – *Dracophyllum* (MD), Mānuka – Heather (MH), and Mānuka – Mānuka (MM).

^i^ Compounds varified by authentic standards


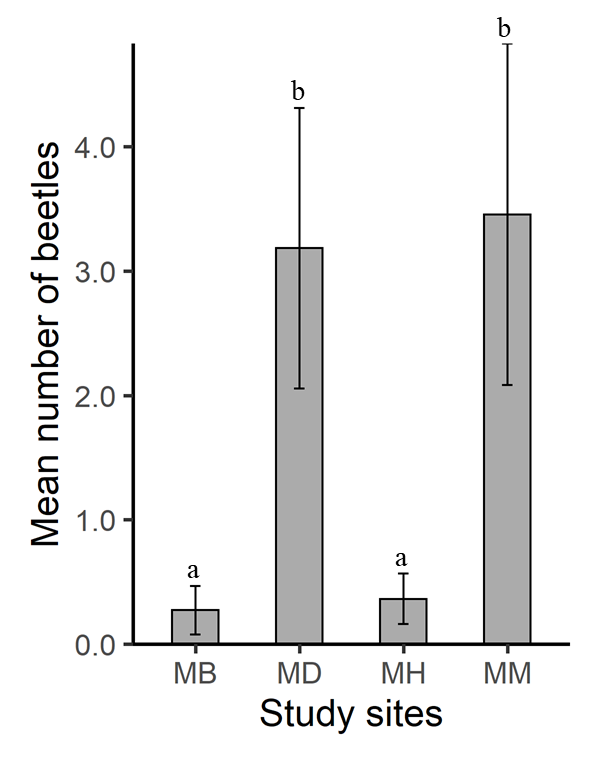


**Figure S1**. Abundance of mānuka beetles between the four study sites. Beetles were captured using in summer the flight interception traps (n = 3), pitfall traps (n = 3) and by directly beating the target plant foliage on a tray (n = 5). Data was analysed using GLM assuming poisson distribution (log-link). **Abbreviations:** Mānuka – Broom (MB), Mānuka – *Dracophyllum* (MD), Mānuka – Heather (MH), and Mānuka – Mānuka (MM). Different letters indicate significant differences (P < 0.05) between sites.
